# Comprehensive Analysis of NAC Domain Transcription Factor Gene Family in *Populus trichocarpa*

**DOI:** 10.1186/1471-2229-10-145

**Published:** 2010-07-15

**Authors:** Ruibo Hu, Guang Qi, Yingzhen Kong, Dejing Kong, Qian Gao, Gongke Zhou

**Affiliations:** 1 Qingdao Institute of BioEnergy and Bioprocess Technology, Chinese Academy of Sciences, Qingdao 266101, PR China; 2 Current address: Complex Carbohydrate Research Center, The University of Georgia, 315 Riverbend Road, Athens, GA 30602, USA

## Abstract

**Background:**

NAC (**NAM, ATAF1/2 **and **CUC2**) domain proteins are plant-specific transcriptional factors known to play diverse roles in various plant developmental processes. NAC transcription factors comprise of a large gene family represented by more than 100 members in *Arabidopsis*, rice and soybean etc. Recently, a preliminary phylogenetic analysis was reported for NAC gene family from 11 plant species. However, no comprehensive study incorporating phylogeny, chromosomal location, gene structure, conserved motifs, and expression profiling analysis has been presented thus far for the model tree species *Populus*.

**Results:**

In the present study, a comprehensive analysis of NAC gene family in *Populus *was performed. A total of 163 full-length NAC genes were identified in *Populus*, and they were phylogeneticly clustered into 18 distinct subfamilies. The gene structure and motif compositions were considerably conserved among the subfamilies. The distributions of 120 *Populus *NAC genes were non-random across the 19 linkage groups (LGs), and 87 genes (73%) were preferentially retained duplicates that located in both duplicated regions. The majority of NACs showed specific temporal and spatial expression patterns based on EST frequency and microarray data analyses. However, the expression patterns of a majority of duplicate genes were partially redundant, suggesting the occurrence of subfunctionalization during subsequent evolutionary process. Furthermore, quantitative real-time RT-PCR (RT-qPCR) was performed to confirm the tissue-specific expression patterns of 25 NAC genes.

**Conclusion:**

Based on the genomic organizations, we can conclude that segmental duplications contribute significantly to the expansion of *Populus *NAC gene family. The comprehensive expression profiles analysis provides first insights into the functional divergence among members in NAC gene family. In addition, the high divergence rate of expression patterns after segmental duplications indicates that NAC genes in *Populus *are likewise to have been retained by substantial subfunctionalization. Taken together, our results presented here would be helpful in laying the foundation for functional characterization of NAC gene family and further gaining an understanding of the structure-function relationship between these family members.

## Background

Transcriptional regulation of gene expression controls many important cellular processes in plants, such as cellular morphogenesis, signaling transduction and environmental stress responses [1]. Transcription factors (TFs) are types of proteins that regulate gene expression by binding to specific cis-acting promoter elements, thereby activating or repressing the transcriptional rates of their target genes [1,2]. Thus, the identification and functional characterization of TFs is essential for the reconstruction of transcriptional regulatory networks [3]. In plants, totally more than 50 different families of TFs have been identified based on bioinformatics analysis so far [1,3]. The *Arabidopsis *genome encodes at least 1550 TFs, accounting for about 6.2% of its estimated total number of gene [4]. As for *Populus*, about 6.4% of its genome was found to encode more than 2900 TFs [3,5].

NAC (**NAM, ATAF1/2 **and **CUC2**) domain proteins comprise of one of the largest plant-specific TFs represented by ~105 genes in *Arabidopsis *[6], ~140 genes in rice [7] and ~101 genes in soybean genome [8]. NAC proteins commonly possess a conserved NAC domain at the N-terminus, which comprises nearly 160 amino acid residues that are divided into five subdomains (A-E) [6,9,10]. A sequence region of 60 residues within the NAC domain contains a unique TF fold consisting of a twisted-sheet bounded by a few helical elements [11]. NAC domain has been implicated in nuclear localization, DNA binding, and the formation of homodimers or heterodimers with other NAC domain proteins [11-16]. In contrast, the C-terminal regions of NAC proteins are highly divergent [6-9,14], and confer the regulation diversities of transcriptional activation activity [11,13,15-22].

NAC proteins have been implicated to participate in a wide range of plant developmental processes, including shoot apical meristem development [10,23-26], floral morphogenesis [27], lateral root development [15,28], leaf senescence [29,30], stress inducible flowering induction [31,32], embryo development [11], cell cycle control [33-35], hormone signaling [15,18,33,36], grain nutrient remobilization [30,37] and shoot branching determination [38]. Particularly, numerous NAC domain proteins have also been implicated in plant abiotic stresses and defense responses such as drought, salinity, cold shock, mechanical wounding and viral infection [18,20,28,36,39-47]. Recently, accumulating evidences indicated that a considerable portion of NAC domain proteins also play crucial roles in the processes of xylogenesis, fiber development, and wood formation in vascular plants [17,19,21,48-55].

In *Arabidopsis*, evidences indicated that a subset of closely related NAC domain proteins including NST1/ANAC043 (NAC Secondary Wall Thickening Promoting Factor 1), NST2/ANAC066, and NST3/SND1(Secondary Wall-associated NAC Domain Protein)/ANAC012 act as master transcriptional switches governing secondary cell wall biosynthesis in a partially redundant manner [52-55]. NST1/ANAC043 and NST2/ANAC066 were shown to function redundantly in secondary cell wall thickening in anther endothecium and induced ectopic secondary wall thickenings in various tissues when expressed ectopically [52,55], whereas NST1 and NST3/SND1/ANAC012 redundantly regulate the secondary wall thickening in inter-fascicular fiber of inflorescence stems and secondary xylem of hypocotyls in *Arabidopsis *[53,54]. SND2/ANAC073 and SND3/ANAC010 have also been shown to function in the formation of secondary cell wall in fibers, and their dominant repression resulted in remarkable reduction in the secondary wall thickening [51]. Interestingly, both of SND2 and SND3 seem to function at downstream of NST1 and NST3/SND1, whereas SND2 was identified as a direct target of NST3/SND1 [51]. VND6 and VND7 (Vascular-related NAC-Domain) have been proposed to be regulators of the formation of vascular vessels in *Arabidopsis *[21,56]. They act as key transcriptional switches regulating the differentiation of metaxylem and protoxylem, respectively, in primary roots [21,56]. Consequently, NSTs may activate the secondary wall biosynthetic program in fibers, whereas VNDs are proposed to specifically regulate secondary wall biosynthesis in vascular vessels [51]. Another *XND1/ANAC104 *(Xylem NAC Domain 1) gene has been revealed to influence the differentiation of tracheary elements and xylem development in *Arabidopsis *by negatively regulating terminal secondary wall biosynthesis and programmed cell death in xylem vessel cells.

Although quite a few NAC TFs have been functionally characterized in model plants *Arabidopsis *and rice, the functions of majority of NAC members remain unknown. Especially in *Populus*, the typical model tree species, there are only very limited reports on the functional characterization of NAC TFs. Recently, Zhong *et al *(2009) reported the molecular cloning and functional characterization of six NAC genes in *Populus *[19]. Among the six NAC genes, WND2B (Wood-Associated NAC Domain Transcription Factors) and WND6B effectively complemented the secondary wall defects in *snd1/nst1 *double mutant, and when ectopically over-expressed in *Arabidopsis*, they induced ectopic deposition of secondary walls. These findings demonstrated that *WND2B *and *WND6B *genes are functional orthologs of *Arabidopsis SND1 *and master switches activating secondary wall biosynthesis during wood formation in *Populus *[19]. Shen *et al *(2009) carried out a genome-wide bioinformatics survey on plant NAC domain TFs and identified a total of 1,232 NAC proteins from 11 different plant species including 148 NAC TFs from *Populus *[57]. However, only sequence phylogeny analysis of *Populus *NAC TFs was performed in their report and no detailed analysis including genome organization, gene structure and expression compendium have been conducted [57]. In the present study, we further performed a genome-wide identification of NAC domain TFs in *Populus *and revealed an expanded NAC family with totally 163 members. Detailed analysis including sequence phylogeny, genome organization, gene structure, conserved motifs and expression profiling was performed. It is noteworthy that a subset of more than thirty NAC genes showed the highest level of transcript abundance in differentiating xylem and cambium tissues. Among them, twenty-five genes were further investigated for their tissue-specific expressions by quantitative real-time RT-PCR (RT-qPCR) analysis. Our results may provide a subset of potential candidate NAC genes for future engineering modification of lignocellulosic biomass characteristics in *Populus*.

## Results and Discussion

### Identification of NAC gene family in *Populus*

The NAC domain genes are plant-specific TFs presented as one of the largest multigene families in *Arabidopsis*, rice, soybean, maize, grape and sorghum etc [3,6,8,57]. Hidden Markov Model (HMM) profile of the Pfam NAC domain (PF02365) was exploited as query to identify the NAC genes in *Populus *genome (release 2.0, http://www.phytozome.net/poplar). Initially, a total of 170 nonredundant putative NAC genes were identified. In an attempt to demonstrate the reliability of the identified genes, keyword search with NAM against NCBI nucleotide database was performed, resulting in 153 members which were all included in the NACs identified above. In comparison to the NAC gene family in PlnTFDB http://plntfdb.bio.uni-potsdam.de/v3.0/[3] and DPTF http://dptf.cbi.pku.edu.cn/[58], in which 167 and 172 members were proposed respectively, the 170 gene loci revealed in the present study were roughly in agreement with the former. Then, the discrepancy loci identified in our studies with the two databases mentioned above were further scrutinized to see if any misannotations were inferred. Among them, sixteen genes may represent putative pseudogenes or incorrect annotations, and manual reannotation was performed to correct and reannotate the putative NACs using online web server FGENESH http://linux1.softberry.com/berry.phtml[59]. In this endeavor, seven sequences encoding only truncated proteins were excluded from further analysis. In addition, we combined the newly released genome annotations with the results inferred by FGENESH to make the annotations more convincing. Finally, all the putative 163 NAC genes were further manually analyzed using InterProScan program http://www.ebi.ac.uk/Tools/InterProScan/ to confirm the presence of NAM domain [60]. In a recently published report, a total of 148 NAC genes were identified in *Populus *by a genome-wide bioinformatics survey [57]. In this study, we further revealed an expanded NAC family in *Populus *with totally 163 members. We designated *Populus *NAC genes as PNAC following the nomenclature proposed in the previous study [6]. The identified NAC genes in *Populus *encode proteins ranging from 117 to 718 amino acids (aa) in length with an average of 342 aa. Remarkably, in most cases, two or more *Populus *NAC genes were found for every ortholog in *Arabidopsis*. The detailed information of NAC family genes in *Populus*, including accession numbers and similarities to their *Arabidopsis *orthologs as well as nucleotide and protein sequences was listed in Table 1 and Additional file 1.

**Table 1:** NAC gene family in *Populus*.

Gene symbol	Gene locus	*Arabidopsis *ortholog locus	*Arabidopsis *locus description	Score	E-value
PNAC001	POPTR_0009s05700.1	AT1G01720.1	ANAC002, ATAF1	179	3e-045
PNAC002	POPTR_0018s10310.1	AT1G01720.1	ANAC002, ATAF1	187	8e-048
PNAC003	POPTR_0017s09630.1	AT3G04070.2	ANAC002, ATAF1	146	2e-048
PNAC004	POPTR_0005s20240.1	AT1G01720.1	ANAC002, ATAF1	392	1e-109
PNAC005	POPTR_0005s07060.1	AT1G01720.1	ANAC002, ATAF1	357	5e-099
PNAC006	POPTR_0002s08150.1	AT1G01720.1	ANAC002, ATAF1	416	1e-117
PNAC007	POPTR_0007s04780.1	AT1G01720.1	ANAC002, ATAF1	366	1e-101
PNAC008	POPTR_0015s14770.1	AT1G12260.1	ANAC007, VND4	374	1e-104
PNAC009	POPTR_0003s11250.1	AT1G12260.1	ANAC007, VND4	436	1e-122
PNAC010	POPTR_0012s14660.1	AT1G12260.1	ANAC007, VND4	340	7e-094
PNAC011	POPTR_0001s00220.1	AT1G12260.1	ANAC007, VND4	449	1e-127
PNAC012	POPTR_0010s13980.1	AT1G25580.1	ANAC008	476	1e-134
PNAC013	POPTR_0008s11550.1	AT1G25580.1	ANAC008	509	1e-144
PNAC014	POPTR_0017s11830.1	AT1G26870.1	ANAC009	299	2e-081
PNAC015	POPTR_0004s12850.1	AT1G26870.1	ANAC009	310	2e-084
PNAC016	POPTR_0004s12850.1	AT1G26870.1	ANAC009	100	9e-022
PNAC017	POPTR_0001s45250.1	AT1G32770.1	ANAC012, SND1, NST3	294	6e-080
PNAC018	POPTR_0011s15230.1	AT1G33060.1	ANAC014	420	1e-117
PNAC019	POPTR_0011s15230.1	AT1G33060.2	ANAC014	431	1e-121
PNAC020	POPTR_0014s07220.1	AT1G33060.2	ANAC014	103	3e-022
PNAC021	POPTR_0002s15550.1	AT1G33060.1	ANAC014	135	9e-032
PNAC022	POPTR_0014s07220.1	AT1G33060.1	ANAC014	109	4e-024
PNAC023	POPTR_0002s15530.1	AT1G33060.2	ANAC014	154	1e-037
PNAC024	POPTR_0012s06340.1	AT1G34190.1	ANAC017	117	2e-027
PNAC025	POPTR_0043s00220.1	AT1G34190.1	ANAC017	140	2e-034
PNAC026	POPTR_0013s10770.1	AT1G34190.1	ANAC017	140	2e-034
PNAC027	POPTR_0043s00220.1	AT1G34190.1	ANAC017	180	4e-046
PNAC028	POPTR_0005s22180.1	AT1G34190.1	ANAC017	441	1e-124
PNAC029	POPTR_0002s06210.1	AT1G34190.1	ANAC017	432	1e-121
PNAC030	POPTR_0014s07210.1	AT4G35580.2	ANAC018, NAM	157	4e-039
PNAC031	POPTR_0014s10350.1	AT4G35580.2	ANAC018, NAM	180	9e-046
PNAC032	POPTR_0014s10350.1	AT4G35580.2	ANAC018, NAM	181	5e-046
PNAC033	POPTR_0002s18310.1	AT4G35580.2	ANAC018, NAM	184	3e-047
PNAC034	POPTR_0002s18320.1	AT4G35580.2	ANAC018, NAM	144	2e-034
PNAC035	POPTR_0002s17700.1	AT4G35580.2	ANAC018, NAM	130	2e-030
PNAC036	POPTR_0002s15560.1	AT4G35580.2	ANAC018, NAM	171	4e-093
PNAC037	POPTR_0014s10320.1	AT4G35580.2	ANAC018, NAM	138	8e-033
PNAC038	POPTR_0014s10320.1	AT4G35580.2	ANAC018, NAM	129	9e-031
PNAC039	POPTR_0010s18180.1	AT1G54330.1	ANAC020	290	9e-079
PNAC040	POPTR_0008s08100.1	AT1G54330.1	ANAC020	316	1e-086
PNAC041	POPTR_0005s10100.1	AT1G56010.2	ANAC021/22, AtNAC1	323	7e-089
PNAC042	POPTR_0007s08420.1 POPTR_0007s08430.1	AT1G56010.2	ANAC021/22, AtNAC1	337	5e-093
PNAC043	POPTR_0004s04920.1	AT1G61110.1	ANAC025	232	3e-061
PNAC044	POPTR_0016s05580.1	AT1G61110.1	ANAC025	137	2e-032
PNAC045	POPTR_0016s08970.1	AT1G61110.1	ANAC025	205	2e-053
PNAC046	POPTR_0006s13140.1	AT1G61110.1	ANAC025	247	7e-066
PNAC047	POPTR_0011s04650.1	AT1G61110.1	ANAC025	339	1e-093
PNAC048	POPTR_0004s03820.1	AT1G61110.1	ANAC025	332	3e-091
PNAC049	POPTR_0017s02020.1	AT1G65910.1	ANAC028	464	1e-130
PNAC050	POPTR_0004s07910.1	AT1G65910.1	ANAC028	465	1e-131
PNAC051	POPTR_0011s12190.1	AT1G65910.1	ANAC028	68	1e-011
PNAC052	POPTR_0014s04080.1	AT1G65910.1	ANAC028	73	1e-013
PNAC053	POPTR_0010s17350.1	AT1G69490.1	ANAC029, ATNAP, NAP	305	2e-083
PNAC054	POPTR_0003s10250.1	AT1G69490.1	ANAC029, ATNAP, NAP	119	3e-027
PNAC055	POPTR_0014s02580.1	AT1G69490.1	ANAC029, ATNAP, NAP	89	4e-018
PNAC056	POPTR_0019s11330.1	AT1G71930.1	ANAC030, VND7	249	9e-067
PNAC057	POPTR_0015s00520.1	AT1G71930.1	ANAC030, VND7	71	1e-012
PNAC058	POPTR_0013s11740.1	AT1G71930.1	ANAC030, VND7	305	2e-083
PNAC059	POPTR_0005s27720.1	AT1G76420.1	ANAC031, CUC3	282	2e-076
PNAC060	POPTR_0002s00730.1	AT1G76420.1	ANAC031, CUC3	319	2e-087
PNAC061	POPTR_0001s22770.1	AT1G77450.1	ANAC032	65	8e-011
PNAC062	POPTR_0008s07950.1	AT1G79580.3	ANAC033	333	1e-091
PNAC063	POPTR_0010s18420.1	AT1G79580.3	ANAC033	344	5e-095
PNAC064	POPTR_0019s09130.1	AT1G79580.3	ANAC033	56	2e-008
PNAC065	POPTR_0003s03670.1	AT2G02450.2	ANAC034, ANAC035	318	2e-087
PNAC066	POPTR_0004s23970.1	AT2G02450.2	ANAC034, ANAC035	310	1e-084
PNAC067	POPTR_0005s10610.1	AT2G17040.1	ANAC036	249	1e-066
PNAC068	POPTR_0004s18860.1	AT2G17040.1	ANAC036	321	4e-088
PNAC069	POPTR_0009s14370.1	AT2G17040.1	ANAC036	315	2e-086
PNAC070	POPTR_0007s13910.1	AT2G18060.1	ANAC037, VND1	428	1e-120
PNAC071	POPTR_0007s13910.1	AT2G18060.1	ANAC037, VND1	432	1e-121
PNAC072	POPTR_0006s29180.1	AT2G24430.2	ANAC038, ANAC039	348	3e-096
PNAC073	POPTR_0018s03910.1	AT2G24430.2	ANAC038, ANAC039	289	2e-078
PNAC074	POPTR_0016s02780.1	AT2G24430.2	ANAC038, ANAC039	65	4e-011
PNAC075	POPTR_0097s00280.1	AT2G24430.2	ANAC038, ANAC039	65	7e-011
PNAC076	POPTR_0009s16290.1	AT2G27300.1	ANAC040, NTL8	272	2e-073
PNAC077	POPTR_0015s00940.1	AT2G33480.1	ANAC041	100	9e-022
PNAC078	POPTR_0017s06000.1	AT2G33480.1	ANAC041	89	3e-018
PNAC079	POPTR_0009s07640.1	AT2G33480.1	ANAC041	59	3e-009
PNAC080	POPTR_0001s11660.1	AT2G43000.1	ANAC042	245	2e-065
PNAC081	POPTR_0005s22710.1	AT2G43000.1	ANAC042	268	2e-072
PNAC082	POPTR_0003s14950.1	AT2G43000.1	ANAC042	245	3e-065
PNAC083	POPTR_0002s05820.1	AT2G43000.1	ANAC042	296	7e-081
PNAC084	POPTR_0014s10060.1	AT2G46770.1	ANAC043, NST1	381	1e-106
PNAC085	POPTR_0002s17950.1	AT2G46770.1	ANAC043, NST1	370	1e-103
PNAC086	POPTR_0011s15640.1	AT2G46770.1	ANAC043, NST1	298	6e-081
PNAC087	POPTR_0001s35490.1	AT3G01600.1	ANAC044	259	2e-069
PNAC088	POPTR_0019s13130.1	AT3G03200.1	ANAC045	87	3e-018
PNAC089	POPTR_0001s26380.1	AT3G04070.2	ANAC047	189	3e-048
PNAC090	POPTR_0013s05080.1	AT3G04070.1	ANAC047	338	4e-093
PNAC091	POPTR_0019s04690.1	AT3G04070.1	ANAC047	332	2e-091
PNAC092	POPTR_0017s05970.1	AT3G04070.2	ANAC047	87	8e-018
PNAC093	POPTR_0006s04990.1	AT3G04070.2	ANAC047	122	3e-028
PNAC094	POPTR_0010s23630.1	AT3G10480.1	ANAC050	418	1e-117
PNAC095	POPTR_0006s02930.1	AT3G10480.2	ANAC050	75	5e-014
PNAC096	POPTR_0097s00260.1	AT3G10480.2	ANAC050	70	1e-012
PNAC097	POPTR_0302s00220.1	AT3G10480.2	ANAC050	74	2e-013
PNAC098	POPTR_0013s07720.1	AT3G10480.2	ANAC050	64	6e-011
PNAC099	POPTR_0006s02910.1	AT3G10480.2	ANAC050	74	2e-013
PNAC100	POPTR_0011s12420.1	AT3G15510.1	ANAC056, AtNAC2	322	2e-088
PNAC101	POPTR_0001s41490.1	AT3G15510.1	ANAC056, AtNAC2	345	4e-095
PNAC102	POPTR_0011s05760.1	AT3G15510.1	ANAC056, AtNAC2	221	1e-058
PNAC103	POPTR_0015s05450.1	AT3G17730.1	ANAC057	372	1e-103
PNAC104	POPTR_0012s03510.1	AT3G17730.1	ANAC057	350	4e-097
PNAC105	POPTR_0015s03700.1	AT3G18400.1	ANAC058	325	3e-089
PNAC106	POPTR_0012s05280.1	AT3G18400.1	ANAC058	315	4e-086
PNAC107	POPTR_0009s02430.1	AT3G44350.2	ANAC061	256	8e-069
PNAC108	POPTR_0001s22630.1	AT3G44350.2	ANAC061	277	5e-075
PNAC109	POPTR_0006s19350.1	AT3G44350.1	ANAC061	81	8e-016
PNAC110	POPTR_0015s00640.1	AT3G49530.1	ANAC062	307	9e-084
PNAC111	POPTR_0012s00760.1	AT3G49530.1	ANAC062	306	2e-083
PNAC112	POPTR_0013s09280.1	AT4G10350.1	ANAC070	436	1e-123
PNAC113	POPTR_0019s09400.1	AT4G10350.1	ANAC070	451	1e-127
PNAC114	POPTR_0003s08830.1	AT4G17980.1	ANAC071	296	1e-080
PNAC115	POPTR_0001s02710.1	AT4G17980.1	ANAC071	296	1e-080
PNAC116	POPTR_0019s13140.1	AT4G17980.1	ANAC071	89	5e-018
PNAC117	POPTR_0009s05690.1	AT4G27410.2	ANAC072, RD26	189	5e-048
PNAC118	POPTR_0011s12400.1	AT4G27410.2	ANAC072, RD26	353	9e-098
PNAC119	POPTR_0007s02050.1	AT4G27410.2	ANAC072, RD26	87	1e-017
PNAC120	POPTR_0001s41460.1	AT4G27410.2	ANAC072, RD26	375	1e-104
PNAC121	POPTR_0017s04800.1	AT4G28500.1	ANAC073	344	4e-095
PNAC122	POPTR_0007s01350.1	AT4G28500.1	ANAC073	337	6e-093
PNAC123	POPTR_0011s05740.1	AT4G28500.1	ANAC073	345	2e-095
PNAC124	POPTR_0004s04900.1	AT4G28500.1	ANAC073	355	2e-098
PNAC125	POPTR_0005s24750.1	AT4G28530.1	ANAC074	271	4e-073
PNAC126	POPTR_0002s03830.1	AT4G28530.1	ANAC074	276	7e-075
PNAC127	POPTR_0006s15740.1	AT4G29230.1	ANAC075	511	1e-145
PNAC128	POPTR_0006s15740.1	AT4G29230.1	ANAC075	486	1e-137
PNAC129	POPTR_0010s23650.1	AT5G04410.1	ANAC078, NAC2	483	1e-136
PNAC130	POPTR_0008s03170.1	AT5G04410.1	ANAC078, NAC2	499	1e-141
PNAC131	POPTR_0007s03840.1	AT5G09330.3	ANAC082	265	4e-071
PNAC132	POPTR_0005s06040.1	AT5G09330.3	ANAC082	256	2e-068
PNAC133	POPTR_0015s11420.1	AT5G13180.1	ANAC083	238	2e-063
PNAC134	POPTR_0001s33260.1	AT5G13180.1	ANAC083	162	1e-040
PNAC135	POPTR_0017s09030.1	AT5G13180.1	ANAC083	151	3e-037
PNAC136	POPTR_0003s16490.1	AT5G13180.1	ANAC083	310	4e-085
PNAC137	POPTR_0001s13380.1	AT5G13180.1	ANAC083	309	1e-084
PNAC138	POPTR_0012s10530.1	AT5G13180.1	ANAC083	228	3e-060
PNAC139	POPTR_0004s11840.1	AT5G17260.1	ANAC086	89	7e-019
PNAC140	POPTR_0012s03100.1	AT5G17260.1	ANAC086	87	4e-018
PNAC141	POPTR_0012s03130.1	AT5G17260.1	ANAC086	95	1e-020
PNAC142	POPTR_0014s10370.1	AT5G17260.1	ANAC086	139	2e-033
PNAC143	POPTR_0012s03120.1	AT5G17260.1	ANAC086	68	6e-019
PNAC144	POPTR_0019s04710.1	AT5G18270.2	ANAC087	267	1e-071
PNAC145	POPTR_0013s05110.1	AT5G18270.2	ANAC087	248	6e-066
PNAC146	POPTR_0016s07680.1	AT5G22380.1	ANAC090	218	3e-057
PNAC147	POPTR_0016s07690.1	AT5G22380.1	ANAC090	213	7e-056
PNAC148	POPTR_0006s22580.1	AT5G22380.1	ANAC090	242	2e-064
PNAC149	POPTR_0097s00210.1	AT5G24590.2	ANAC091, TIP	56	3e-008
PNAC150	POPTR_0017s08490.1	AT5G39820.1	ANAC094	50	3e-006
PNAC151	POPTR_0011s11600.1	AT5G53950.1	ANAC098, CUC2	311	3e-085
PNAC152	POPTR_0001s40680.1	AT5G53950.1	ANAC098, CUC2	300	1e-081
PNAC153	POPTR_0017s12210.1	AT5G61430.1	ANAC100, ATNAC5	377	1e-105
PNAC154	POPTR_0015s02170.1	AT5G61430.1	ANAC100, ATNAC5	446	1e-125
PNAC155	POPTR_0012s01610.1	AT5G61430.1	ANAC100, ATNAC5	448	1e-126
PNAC156	POPTR_0006s24740.1	AT5G62380.1	ANAC101, VND6	61	1e-009
PNAC157	POPTR_0004s10720.1	AT5G62380.1	ANAC101, VND6	74	9e-014
PNAC158	POPTR_0005s08440.1	AT5G62380.1	ANAC101, VND6	74	2e-013
PNAC159	POPTR_0014s16160.1	AT5G62380.1	ANAC101, VND6	59	6e-009
PNAC160	POPTR_0007s04250.1	AT5G64530.1	ANAC104, XND1	185	2e-047
PNAC161	POPTR_0003s01720.1	AT5G64530.1	ANAC104, XND1	231	2e-061
PNAC162	POPTR_0001s21440.1	AT5G64530.1	ANAC104, XND1	215	1e-056
PNAC163	POPTR_0005s06510.1	AT5G64530.1	ANAC104, XND1	199	1e-051

Gene loci are from the Phytozome website http://www.phytozome.net/, release 2.0). A complete list of the coding sequences (CDS), deduced amino acid sequences and genomic DNA sequences is available in Additional file 1.

The NAC gene family in *Populus *is by far the largest one compared to the estimates for other plant species, which range from ~105 in *Arabidopsis *[6], ~140 in rice [7] and ~101 in soybean [8]. The member of NAC genes in *Populus *is roughly 1.58 fold than that of *Arabidopsis*, which is in consistency with the ratio of 1.4~1.6 putative *Populus *homologs for each *Arabidopsis *gene based on comparative genomics studies [5]. In comparison to its closest woody perennial grape (*Vitis vinifera*), which possesses only about 79 NAC genes [3], the NAC genes in *Populus *seems to be highly expanded [5]. It can be speculated that the presence of more NAC genes in *Populus *genome may reflect the great needs for these genes to involve in the complicated transcriptional regulations in this woody perennial species. This expansion appears to be arisen from multiple gene duplication events, including a whole-genome duplication event in the *Populus *lineage followed by multiple segmental and tandem duplication events [5].

### Phylogenetic analysis of NAC gene family in *Populus*, *Arabidopsis *and rice

**Figure 1:**
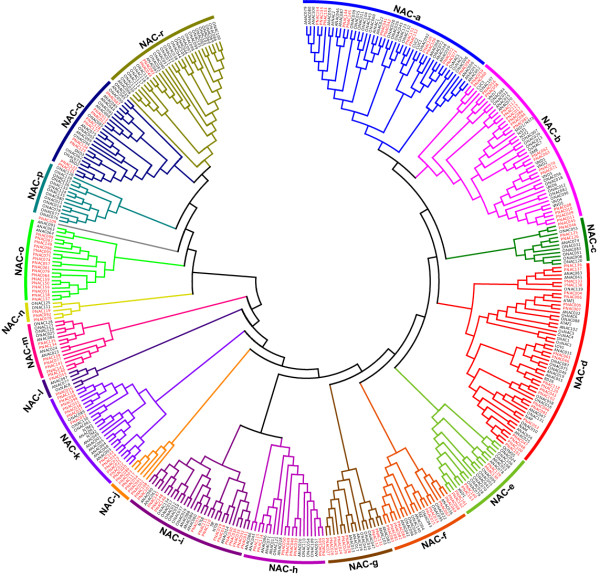
**Joined phylogenetic tree of NAC domain-containing proteins from *Populus*, *Arabidopsis *and rice**. The deduced full-length amino acid sequences of 163 *Populus*, 105 *Arabidopsis *and 140 rice NAC genes were aligned by Clustal X 1.83 and the phylogenetic tree was constructed using MEGA 4.0 by the Neighbor-Joining (NJ) method with 1,000 bootstrap replicates. Each NAC subfamily was indicated in a specific color. Members of NAC protein from *Populus *were denoted in red.

To examine the phylogenetic relationship among the NAC domain proteins in *Populus*, *Arabidopsis *and rice, an unrooted tree was constructed from alignments of the full-length NAC protein sequences (Figure 1). The phylogenetic tree was constructed using MEGA 4.0 by employing the Neighbor-Joining (NJ), Minimal Evolution (ME) and Maximum Parsimony (MP) methods, respectively. The tree topologies produced by the three algorithms were largely comparable with only minor modifications at interior branches (data not shown). Therefore, only the NJ phylogenetic tree was subject to further analysis in our study. Moreover, we constructed the phylogenetic tree with the conserved N-terminal NAC domains A to E using the same algorithm, which was largely consistent to the phylogenetic analyses performed by the previous studies [57] (see details in Additional file 2). As indicated in Figure 1, the phylogenetic tree divided the NAC family proteins into 18 distinct subgroups. PNAC109 was distinguished from other NAC members and formed an individual clade with a robust bootstrap value support (99%) (Figure 1 and Additional file 3). For simplicity, the subgroups were designated as alphabetical families (NAC-a to NAC-r) based on tree topologies. Although the bootstrap values were somewhat low due to the large number of sequences, which was also presented in previous studies [61-65], more significant bootstrap values in the distal branches allowed us to group the *Populus*, *Arabidopsis *and rice NAC proteins into distinct families (Additional file 3). Moreover, we sought other evidences such as gene structure, motif compositions and expression patterns as described below to support the reliability of the subgroup classification.

In general, the NAC members demonstrated an interspersed distribution in majority of subfamilies, indicating that the expansion of NAC genes occurred before the divergence of *Populus*, *Arabidopsis *and rice. Noticeably, NAC genes with same functions showed a tendency to fall into one subgroup, similar to the previous reports [7,8,57]. For instances, subfamily NAC-a encompassed the NAC proteins involved in shoot organ boundary delimitation (eg. CUC1, 2) [25,26], whereas subfamily NAC-d possessed all the NAC proteins that function in shoot apical meristem (SAM) establishment, pattern formation in embryos and flowers (eg. NAM) [11], stress responses (eg. ATAF1, RD26 and OsNAC6) [18,47,66] and leaf senescence (eg. NAP) [29]. This group also encompassed ANAC019 and ANAC055, which have been shown to be induced by abiotic stresses (ABA, drought and salinity) and to enhance tolerance to drought when ectopically over-expressed [40]. The membrane-associated NAC proteins that mediate either cytokinin signaling during cell division or endoplasmic reticulum stress responses were clustered into the NAC-i and NAC-k subfamilies. The members in these two subgroups are distinct with other NACs in that they harbor a transmembrane (TM) motif with a predicted α-helix in the far C-terminal region. In NAC-k subfamily, there were only two well functionally characterized members namely NTM1 and NTM2. The well-characterized genes in subfamily NAC-f included FEZ, which was demonstrated to be associated with the orientation of cell division in root stem cells [34].

Remarkably, subfamily NAC-j did not include any *Arabidopsis *and rice NAC proteins but only members from *Populus*, suggesting that they may have been either lost in *Arabidopsis *and rice or acquired in the *Populus *lineages after divergence from the last common ancestor. We speculate that these subsets of genes may also have specialized roles with respect to the woody perennial habit in *Populus*. Phylogenetic analysis also revealed a subgroup NAC-o that contained sequence representatives in *Populus *and *Arabidopsis *but not in rice, indicating that the NAC members in this subgroup were acquired or differentially retained in eudicots post-divergence from monocots. In contrast, the subgroup NAC-p was composed of 13 rice NAC proteins, but no *Arabidopsis *and *Populus *proteins, which suggests diversification and expansion of this subgroup after the monocot-eudicot radiation.

The NAC proteins associated with secondary wall formation in fiber and vascular vessel development were divided into three independent subfamilies. All NSTs (NST1, NST2, NST3/SND1) and VNDs (VND1-VND7) were clustered into one subfamily NAC-b, whereas SND2, SND3 and their counterparts were grouped into NAC-q. The other well-characterized NAC member XND1 in secondary wall formation was assigned to another different subfamily (NAC-e). All the six functional characterized NST3/SND1 orthologs (PtWND1B to 6B) in *Populus *fell into subfamily NAC-b [19]. Although enormous evidences exemplified that all these NAC genes performed as key transcriptional switches in the secondary cell wall formation process, their exact functional roles diversified [52-55]. The phylogenetic analysis conducted herein may provide a potential supporting for their functional diversities. What's more, the tissue-specific expression profiling available on GENEVESTIGATOR lent further supports for this notion [67]. The expression pattern of XND1 in *Arabidopsis *was extremely different from that of NSTs and VNDs. Nevertheless, the expression pattern of SND2 and SND3 was somewhat more similar to that of SND1/NST3 and NST2 (Additional file 4). In accordance with the recent report, NST, VND, SND, and XND from 11 different species were also classified into three distinct phylogenetic subgroups conducted by Maximum Likelihood (ML) algorithm [57]. Another well-characterized gene namely SOMBRERO (SMB), which has been shown to control the reorientation and timing of cell division in root stem cells by negatively regulating FEZ activity [34], was clustered together with NSTs and VNDs into subfamily NAC-b. We inspected the expression patterns of SMB on GENESTIGATOR [67] but failed in an attempt to find any clues for their diversified roles in secondary wall formation. SMB was specifically highly expressed in callus and lateral root cap rather than secondary cell wall enriching tissues, suggesting a specific role in cell division and root development (Additional file 4).

It is noteworthy that the number of *Populus *genes was generally overrepresented than that of *Arabidopsis *and rice in almost all clades, especially within NAC-o subfamily, in which *Populus *NAC genes was particularly overrepresented and almost sixfold the number of genes with respect to *Arabidopsis*. Alternatively, in subfamily NAC-a and q, the number of *Arabidopsis *and rice genes nearly equaled that of *Populus*. In contrast, the number of rice NAC genes in subfamily NAC-r showed an overwhelming predominance with respect to that of *Populus*.

The phylogenetic tree obtained in this study is largely consistent with previous analyses [6-8,57], although there are some discrepancies. The first systematic analysis of *Arabidopsis *and rice NAC proteins classified them into 18 subgroups [6]. However, another phylogenetic analysis of rice NAC proteins suggested that the NAC family can be divided into five groups and each subfamily was largely diversified [7]. In a report concerning soybean NAC family, the genes were clustered into 15 distinct subfamilies with robust bootstrap supporting [8]. More recently, systematic phylogenetic analyses of numerous NAC proteins from a wide range of plant species indicated that NAC proteins can be divided into eight subfamilies [57]. In our opinions, the main reason for the discrepancies of the phylogenetic trees reported may lie in the fact that all the previous NAC protein classifications were based on the conserved N-terminal NAC domains, either from sub-domain A to D or A to E, which did not take the highly divergent C-terminal sequences into consideration [6-8,57]. In an attempt to gain better understanding of the phylogeny of NAC gene family, we performed the phylogenetic analysis with inclusion of the highly diverse C-terminal sequence. Moreover, different algorithms exploited in the phylogenetic analyses may lead to the inconsistent interpretations. In the previous analyses, different algorithms including Neighbor-Joining (NJ) [6,8], Maximum likelihood (ML) [57] and Bayesian method [7] were implemented, which may make the results less comparable.

Inspection of the phylogenetic tree topology revealed several pairs of NAC proteins with a high degree of homology in the terminal nodes of each subfamily, suggesting that they are putative paralogous pairs (homologous genes within a species that diverged by gene duplication) (Figure 1 and Additional file 3). Totally, forty-nine pairs of putative paralogous NAC proteins were identified, accounting for more than 60% of the entire family, with sequence identities ranging from 71% to 97% (see Additional file 5 for details).

### Chromosomal location and gene duplication of *Populus *NAC genes

**Figure 2:**
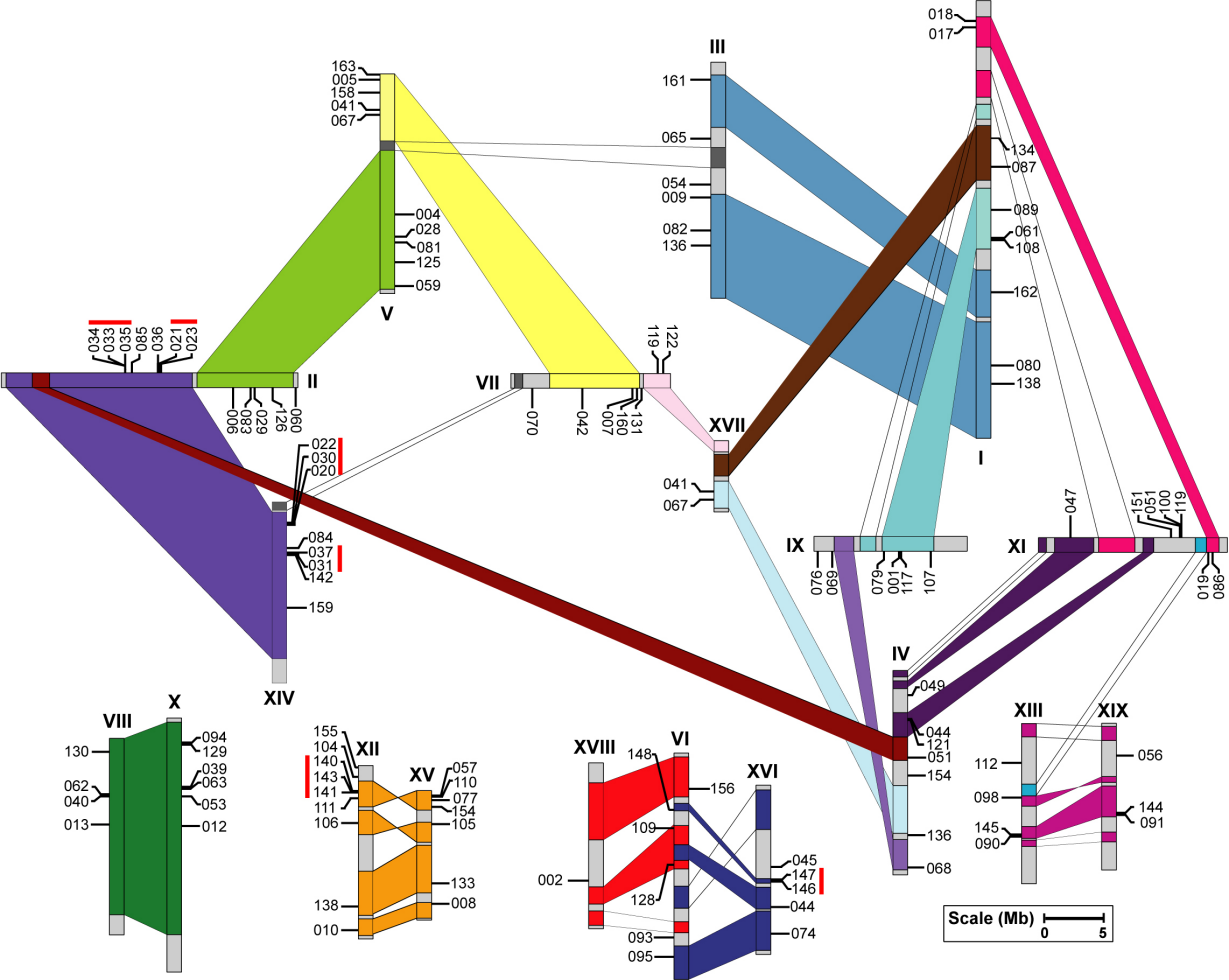
**Chromosomal locations of *Populus *NAC genes**. Only 120 NAC genes are mapped to the 19 Linkage Groups (LG), while the other 43 genes reside on unassembled scaffolds. The schematic diagram of genome-wide chromosome organization arisen from the salicoid genome duplication event in *Populus *was adapted from Tuskan *et al*., (2006) [5]. Segmental duplicated homeologous blocks are indicated with the same color. Only the duplicated regions containing NAC genes are connected with lines in shaded colors. Tandemly duplicated genes are indicated with red lines. Scale represents a 5 Mb chromosomal distance.

*In silico *mapping of the gene loci showed that 163 *Populus *NAC genes were distributed across all 19 Linkage Groups (LG). In the currently released sequences, totally 120 NAC genes were mapped to LGs, whereas 43 genes were remained on as yet unmapped scaffolds. The distributions of *Populus *NAC genes across the LGs appeared to be non-random (Figure 2). LG II encompassed the largest number of 12 NAC genes followed by 10 on LG V and LG I, respectively. In contrast, only one NAC gene was found on LG XVIII and two NAC genes were on LG XVII. Substantial clustering of *Populus *NAC genes was evident on several LGs, especially on those with high densities of NAC genes. For instances, PNAC020, 022 and 030 were cluster localized on a 17 kb segment on LG XIV, and three NACs (PNAC033, 034 and 035) were organized in another cluster within a 10 kb fragment on LG II, whereas PNAC140, 141 and 143 were arranged in a cluster localized to a 11 kb segment on LG XII (Figure 2).

Previous studies revealed that *Populus *genome had undergone at least three rounds of genome-wide duplications followed by multiple segmental duplication, tandem duplication, and transposition events such as retroposition and replicative transposition [5,68]. Among them, the segmental duplication associated with the salicoid duplication event that occurred 65 million years (Ma) ago significantly contribute to the amplification of many multi-gene families [61,69-71]. To determine the possible relationship between the NAC genes and potential segmental duplications, we mapped *Populus *NACs to the duplicated blocks established in the previous studies [5]. The distributions of NAC genes relative to the corresponding duplicate genomic blocks were illustrated in Figure 2. Within the 36 identified duplicated blocks associated with the recent salicoid duplication event, about 73% (87 of 120) of *Populus *NACs were preferentially retained duplicates that locate in both duplicated regions of 28 block pairs, whereas nine block pairs only harbored NACs on one of the blocks and lack the corresponding duplicates, suggesting that dynamic changes may have occurred following segmental duplication, leading to loss of some of the genes. In contrast, only a small number of 19 NAC genes were located outside of any duplicated blocks.

In addition, the tandem duplications might also have an impact on the expansion of the NAC gene family. Fine mapping analysis revealed the presence of 13 pairs of adjacent genes within a 20 kb distance possibly due to tandem duplication either in inverse or same orientation (Figure 2). It is intriguing that except for one pair (PNAC110 and 119), all the other putative tandem duplicates were represented within the recent segmental duplicate blocks. Similar results have also been reported in several other *Populus *gene families [71,72]. These 26 NAC genes (16%) were represented in 12 distinct tandem duplicate gene clusters, with ten clusters containing two tandem genes and two clusters possessing three tandem genes. But further analysis of their similarities demonstrated that most of the tandem clustered NAC pairs shared relative low similarities (mostly below 50%), which may exclude them from tandem duplicate candidates. Therefore, ultimately only six pairs of NACs were conformed to the standards as tandem duplicates, which were represented in tandem clusters of two to three genes each. These tandem duplicated NAC genes were organized onto duplicated blocks, implying that the occurrence of local duplications was prior to the chromosomal segment duplication.

As a considerable proportion of the NAC proteins appeared to be paralogous pairs as revealed in the phylogenetic analysis, we further investigated whether traceable genome duplication events have contributed to the expansion of the NAC family. Nineteen out of 49 homologous pairs remained in conserved positions on segmental duplicated blocks (Figure 2), suggesting that these genes may be the result of segmental duplication event during the evolution. For 26 homologous pairs, no traceable duplication events could be inferred, because they are mapped to the yet non-assembled scaffolds. Among them, a total of 11 genes were located on segmental duplication blocks with their counterparts not mapped to LGs yet. Meanwhile, a subset of eight NAC genes were located outside of the segmental duplication blocks while their corresponding members mapped to non-assembled scaffolds. Taken together, nine genes of the homologous pairs were definitely not located on any duplicated blocks, and only three of them had the homologous counterparts located on the duplicated blocks. Duplicates of these genes appeared to have lost from the *Populus *genome. Interestingly, one homologous pair PNAC121 and 122 was located on two divergent rather than homologous duplicated blocks, thus we could not figure out any traceable duplication history for this pair of NACs even though they were covered by duplicated blocks.

Based primarily on the genomic organization of NAC genes, we could conclude that segmental duplications exclusively contributed to the complexity of NAC gene family in the *Populus *genome. Similarly, segmental duplications have also been shown to contribute to the expansion of other gene families in *Populus *[61,70-72]. Our studies indicated that *Populus *NAC genes have been preferentially retained at a rate of 73%, which is much higher than the average rate following the salicoid genome-wide duplication and rearrangement events [5]. On a genome-wide scale, approximately one third of predicted genes are retained in duplications resulting from the salicoid duplication event [5]. The high retention rate of duplicated genes was also previously reported in other *Populus *gene families [70,71]. These findings are also in consistency with the previous studies demonstrating that genes involved in transcription regulation and signal transduction are preferentially retained [73-75]. Another plausible explanation to the relatively high retention rate of duplicate genes in NAC gene family may lie in the fact that *Populus *genome has been indicated to evolve at a much slower rate compared to that of *Arabidopsis *[5]. The duplicated genes may undergo divergent fates during subsequent evolution such as nonfunctionalization (loss of original functions), neofunctionalization (acquisition of novel functions), or subfunctionalization (partition of original functions) [76,77]. Whether the duplicated NAC genes correspond to genetic redundancy or have evolved divergent functions remains to be further functionally characterized.

The tandem duplication ratio of NAC genes in this study is relatively lower compared to some other gene families in *Populus*, which was represented in a considerable higher proportion [71,72]. Interestingly, a similar low retention rate of tandem duplicates was also observed for *Populus *ARF gene family [71].

### Gene structure and conserved motifs of *Populus *NAC genes

**Figure 3:**
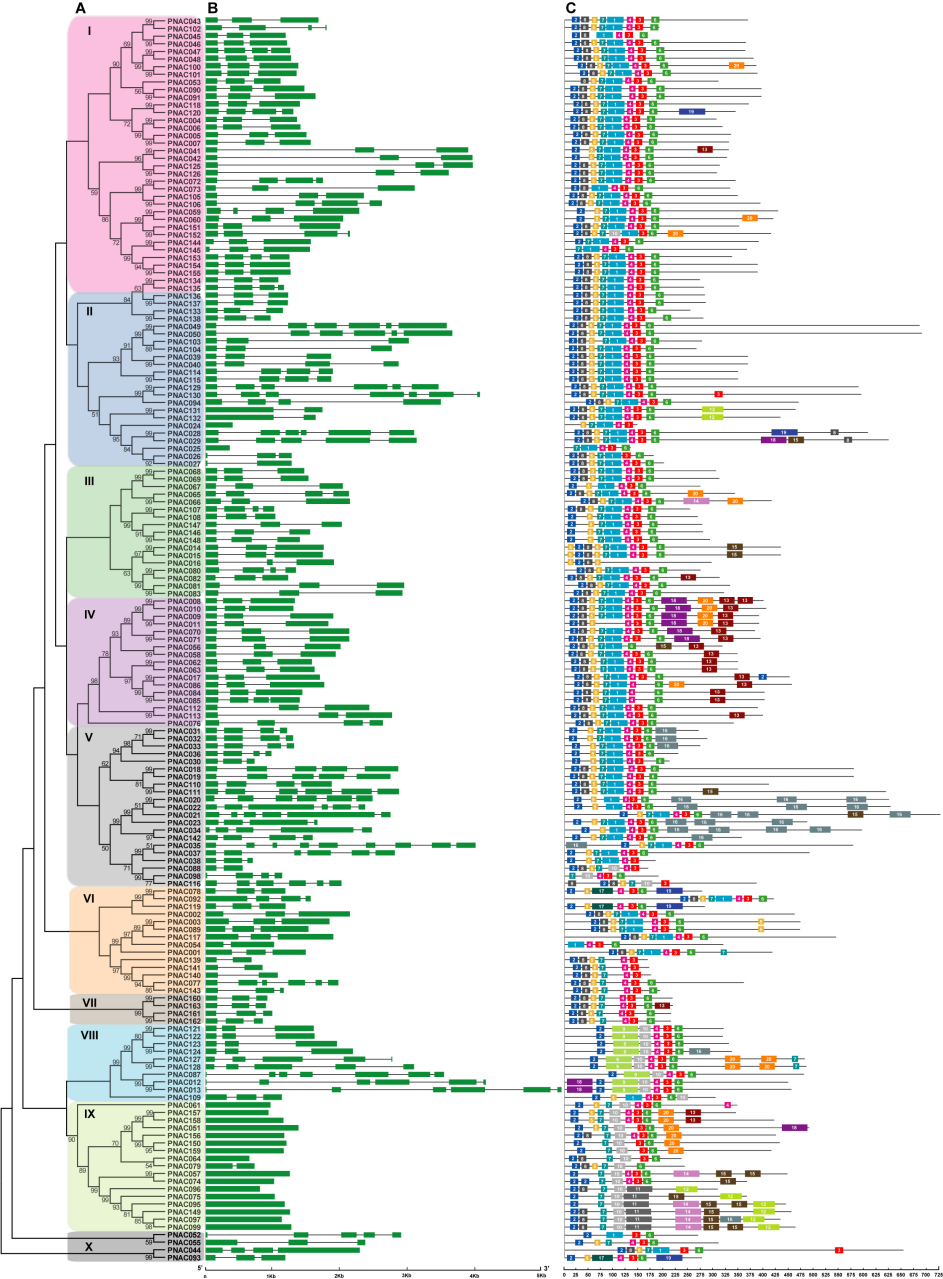
**Phylogenetic relationships, gene structure and motif compositons of *Populus *NAC genes**. **A**. Multiple alignments of 163 full-length amino acids of NAC genes from *Populus *were executed by Clustal X 1.83 and the phylogenetic tree was constructed using MEGA 4.0 by the Neighbor-Joining (NJ) method with 1,000 bootstrap replicates. The percentage bootstrap scores higher than 50% are indicated on the nodes. The ten major phylogenetic subfamilies designated as I to X are marked with different color backgrounds. **B**. Exon/intron structures of NAC genes from *Populus*. Exons and introns are represented by green boxes and black lines, respectively. The sizes of exons and introns can be estimated using the scale at bottom. **C**. Schematic representation of the conserved motifs in the NAC proteins from *Populus *elucidated by MEME. Each motif is represented by a number in the colored box. The black lines represent the non conserved sequences. Refer to Additional file 7 for the details of individual motif.

It is well known that gene structural diversity is a possible mechanism for the evolution of multigene families. In order to gain further insights into the structural diversity of NAC genes, we compared the exon/intron organization in the coding sequences of individual NAC genes in *Populus*. A detailed illustration of the exon/intron structures was shown in Figure 3B. In addition, a separate phylogenetic tree was generated from complete protein sequences of all the NAC genes in *Populus*, which divided the NAC genes into ten subfamilies (Figure 3A). In general, most closely related members in same subfamilies shared similar exon/intron structure in terms of intron number and exon length (Figure 3B). For instances, the NAC genes in subfamily I, III, IV and VII contained two to three introns while those in subfamily IX all possessed no introns with exception of PNAC079, which harbored one intron. In contrast, the gene structure appeared to be more variable in subfamilies II and V, which had the largest number of exon/intron structure variants with striking distinctions. Interestingly, although the exon/intron organization of NACs varied significantly in terms of intron number, the intron phase was remarkably high conserved (Additional file 6), which was indicative of exon shuffling during the evolution process [78].

We further analyzed the exon/intron structure of the paralogous pairs in NAC gene family to obtain traceable intron gain or loss information about these genes. Of the 49 paralogous pairs, thirty-four pairs showed conserved exon/intron structure either in terms of intron numbers or gene length (Figure 3B). Remarkably, twenty-five pairs of genes exhibited a highly conserved distribution of exons and introns, with all members having three exons and two introns. Despite a majority of these genes have arisen from segmental duplication events, the others were located either on non-duplicated blocks or on not-yet assembled scaffolds. While the gene structure was conserved between some paralogous pairs, others exhibited some extent of variation. For example, eleven NAC genes out of the 49 paralogous pairs possessed three exons in their coding regions, whereas their homologous counterparts all contained four exons within nearly identical gene lengths (Figure 3B). We could firmly speculate that the differences in the last exons are probably derived from single intron loss or gain occurred during the process of structural evolution of NAC paralogs. The conserved exon/intron architecture shared by these homologous gene pairs may reciprocally lend supports for the results from the phylogenetic analysis and the duplication events.

To further reveal the diversification of NAC genes in *Populus*, putative motifs were predicted by the program MEME (Multiple Expectation Maximization for Motif Elicitation), and 20 distinct motifs were identified. As expected, most of the closely related members in the phylogenetic tree had common motif compositions, suggesting functional similarities among the NAC proteins within the same subfamily (Figure 3C). However, the biological significance of most of the putative motifs remains to be elucidated as they do not have homologs when searching against Pfam and SMART (Simple Modular Architecture Research Tool) databases [79,80]. The details of the 20 putative motifs were referred in Additional file 7. As illustrated in the previous studies, most of the NAC proteins possessed A to E subdomains in the N termini that conferred the DNA-binding activities. In this study, motif 2, 5, 1, 3 and 6 specifying the NAM subdomain A to E respectively were present in most of the NAC family members in *Populus*, whereas a small subset of sequences did not have all five motifs in the typical NAC DNA-binding domains. Even though the C-terminal regions of NACs were highly divergent, we could also identify at least eight conserved motifs using the MEME motif search tool. Noticeably, some specific motifs were present in NACs from specific subfamilies, for instance, motif 16 for subfamily V, motif 9 for subfamily VIII and motif 11 for subfamily IX. Whether these motifs confer unique functional roles to NACs remains to be further investigated.

Of the 49 paralogous pairs, thirty-seven pairs of NACs shared conserved motif composition with each other, indicative of functional similarities. In contrast, specific motifs located in the C termini were observed for the rest 12 pairs of NACs, for example, motif 13 for PNAC041, 082, 113, 163, motif 14 for PNAC066, and motif 19 for PNAC120. We attempt to speculate that these specific motifs may by some extent attribute to the subfunctionalization or neofunctionalization of the duplicated genes during subsequent evolution processes.

The similar gene structures and conserved motifs of NAC genes in same subfamilies may provide additional supports to the phylogenetic analysis. On the other hand, the differences between gene organization and the divergences in motif compositions among different subfamilies may also indicate that *Populus *NACs are functionally diversified.

### Differential expression profile of *Populus *NAC genes

**Figure 4:**
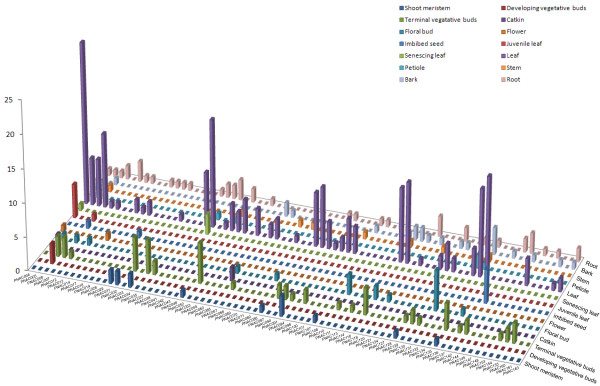
***In silico *EST analysis of *Populus *NAC genes**. EST frequency of 74 *Populus *NAC genes was obtained by screening the EST datasets from various libraries across a set of 14 tissues. Expression of NAC genes was plotted as counts of corresponding ESTs for particular gene in the database.

**Figure 5:**
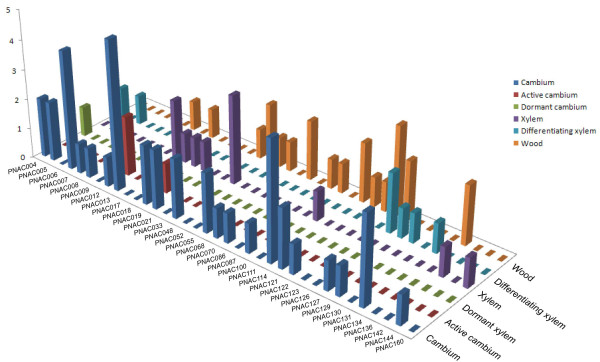
**EST profiles of *Populus *NAC genes in wood-forming tissues**. EST distribution of 36 *Populus *NAC genes was obtained by searching the EST datasets originated from libraries of wood-forming tissues. Expression of NAC genes was plotted as counts of corresponding ESTs for particular gene in the database.

Publicly available Expressed Sequence Tags (ESTs) was considered as a useful means of studying gene expression profiles (digital northern) [81]. A scrutiny of the frequency of ESTs in different databases allows preliminary analysis of gene expression under various conditions and across tissues. The digital expression profiles of 81 *Populus *NAC genes were obtained from EST databases at NCBI (Oct., 2009), while the rest of other 82 NAC genes did not have corresponding EST sequences in the database. Noticeably, the frequency of ESTs was low with most of NACs represented by a single EST sequence, which is consistent with the commonly low transcript abundance feature of transcription factor genes [61]. Thus we postulate that the absent genes in EST database might either transcribe at relatively too low abundance to be detected or had special temporal and spatial expression patterns not examined in the libraries. Another possible explanation is that these genes might be pseudogenes, as they only possessed partial NAC domains as described previously (Figure 3C). In order to gain a better portrait of NAC gene expression profiles, we further largely divided the EST data into two groups, with one group contained all wood-forming tissues and the other derived from the other tissues. The EST digital expression profiles were illustrated in Figure 4, Figure 5 and Additional file 8. The expression profiles demonstrated that majority NACs have rather broad expression patterns with presence in diverse libraries. Not surprisingly, a considerable subset of genes was preferentially expressed in leaves (Figure 4). In addition to leaves, more than 40% (36 of 81) of the NAC genes were especially expressed during wood formation (in cambial zone and xylem) (Figure 5).

It is noteworthy that several putative NAC orthologs between *Arabidopsis *and *Populus *showed strikingly consistent expression patterns, which lend further supporting to the existence of functional conservation between these two species. For example, the expression profiles of homologous pair PNAC031 and 032, which had two corresponding ESTs derived from shoot meristem respectively (Figure 4), further reinforced the notion that they are the closest orthologs of NAM in *Arabidopsis *[11]. The EST data revealed that PNAC013 gene was specifically expressed in active cambium with two detectable ESTs and highly resembled the expression pattern of its closest ortholog ANAC009 in *Arabidopsis*, which was expressed predominantly in highly dividing and expanding tissues such as callus and root cap (Additional file 4).

**Figure 6:**
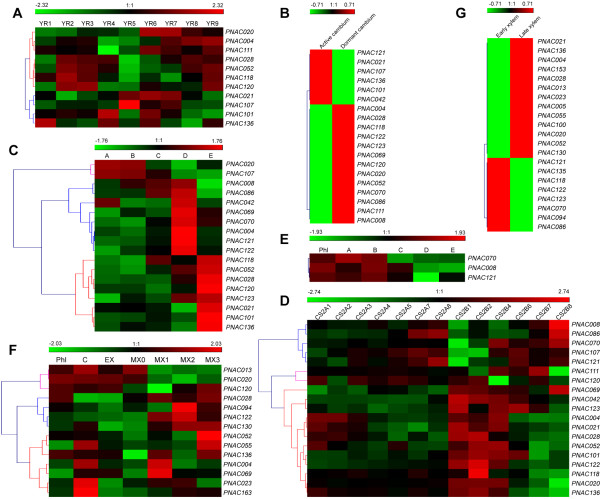
**Expression profiles of *Populus *NAC genes in wood-forming tissues**. Microarray data were obtained from PopGenIE ftp://aspnas.fysbot.umu.se/[84]. The expression data were gene-wise normalized and hierarchical clustered with average linkage. Each row corresponds to the relative expression levels normalized against the maximum value. Color scale at the top of each dendrogram represents relative expression levels: green represents low level and red indicates high level. **A**. Dynamic expression levels of 11 NAC genes in different states of cambiums. YR1 to YR9 represent the nine sampling time points from 20 April (before bud break) until 13 December (cambium dormant). **B**. Expression patterns of 19 NACs in active and dormant cambiums. **C, D**. Hierarchical clustering of expression profiles of 19 NAC genes in the cambial region. Phl, phloem; A, meristematic cells; B, early expansion; C, late expansion; D, secondary wall formation; E, late cell maturation. CS2A1, CS2A2, CS2B1, CS2B2 and CS2B4, phloem; CS2A3, CS2A4, CS2B6 and CS2B7, cambium region; CS2A5, CS2A7, CS2A8 and CS2B8, xylem. **E**. Expression pattern of three NAC genes in cambium regions. The tissues are the same as depicted in C and D. **F**. Expression profiles of 14 NACs in vascular tissues. Phl, phloem; C, cambium; EX, expanding xylem; MX0, initiation of secondary cell-wall deposition stage; MX1-3, maturing xylem. **G**. Deferential expressions of 21 NAC genes during the xylem differentiation. EX, early xylem; LX, late xylem.

Although the digital EST expression analyses provided a first glimpse of the patterns of NAC expression in *Populus*, we could not draw conclusive inferences regarding NAC gene expression due to the obvious limitations of EST data such as relative narrow sample coverage and biases towards highly expressed genes. Thus, we performed a more comprehensive microarray analyses for NAC gene expression profiles. To gain more insight into the expression patterns of NAC genes, a comprehensive expression analysis was further performed by utilizing the publicly available microarray data for *Populus*. Firstly, we re-analyzed the collections of microarray data from wood-formation series available at PopGenIE, a database repository of transcriptomics data available for *Populus *[82,83]. Distinct expression profiles were identified for a total of 29 NAC genes (Figure 6 and Additional file 9), whereas no corresponding probe sets were available for the remaining NACs in the datasets. The relative low coverage of NACs in the datasets analyzed maybe partially due to the fact that these arrays were mainly derived from ESTs rather than offered a whole genome coverage [84].

Two subset of NAC genes exhibited specifically high transcript accumulation in active and dormant cambiums respectively, from two independent microarray datasets [85,86], strongly indicating their specific roles in wood-formation (Figure 6A and 6B). In addition, by incorporating the different microarray data, we were capable to identify different subsets of NAC genes displaying specifically high expression levels in phloem, differentiating xylem and mature xylems, respectively. The expression levels of five NAC genes including PNAC070, 086, 121 and 122 were peaked exclusively in differentiating xylems supported by two independent microarray datasets (Figure 6C and 6G) [87,88]. Among them, PNAC070 (PtWND5A), PNAC086 (PtWND1A) and PNAC008 (PtWND3A) were demonstrated in previous studies as putative redundant homologs involved in *Populus *wood formation [19]. Interestingly, several genes such as PNAC013, 020 and 120 were preferentially expressed in tissues enriched of secondary cell walls, whereas a subset of genes including PNAC028, 052, 055 and 023 showed constitutively high expressions in both secondary and primary cell wall enriching tissues (Figure 6A-E). Similarly, several genes such as PNAC118 and 136 displayed varied peak abundance of transcript across the same tissues tested among different microarray datasets (Figure 6A-E).

**Figure 7:**
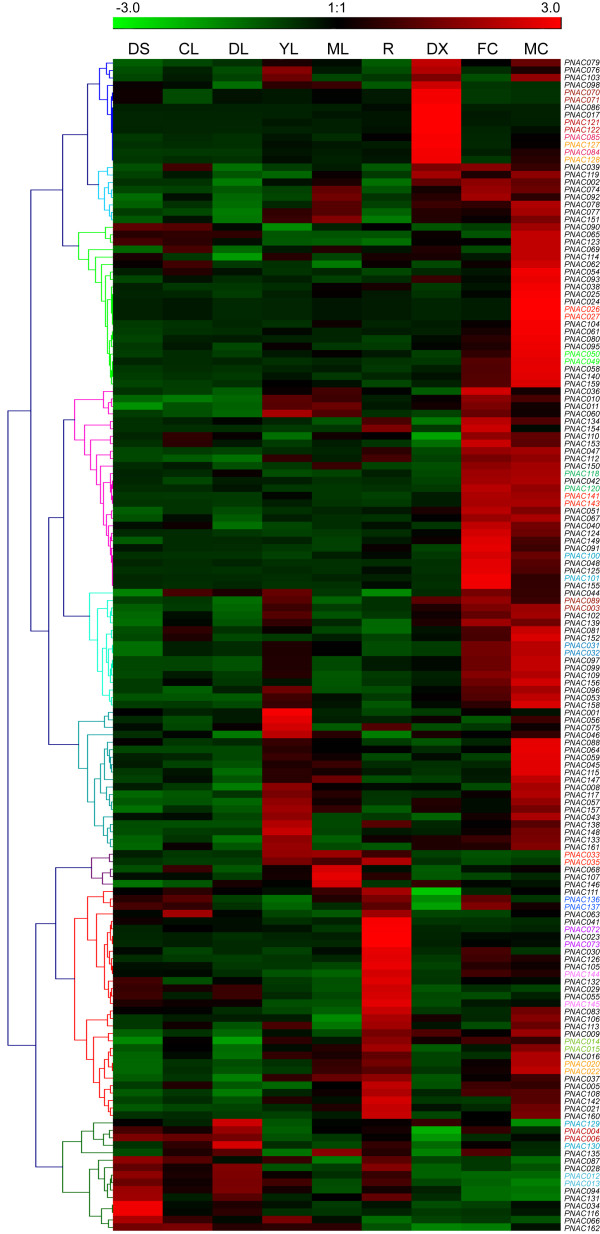
**Hierarchical clustering of expression profiles of *Populus *NAC gene across different tissues**. The genome-wide microarray data generated by Wilkins and coworker were re-analyzes [61]. The expression data were gene-wise normalized and hierarchical clustered based on Pearson correlation. The genes marked in the same color indicate duplicated gene pairs. The relative expression level of particular gene in each row is normalized against the maximum value. Color scale at the top of each dendrogram represents log2 expression values, green represents low level and red indicates high level of transcript abundances. CL, continuous light-grown seedling; DL, etiolated dark-grown seedling transferred to light for 3h; DS, dark-grown seedlings; YL, young leaf; ML, mature leaf; R, root; DX, differentiating xylem; FC, female catkins; MC, male catkins.

As the previous analyzed microarray are mainly derived from ESTs and only a small fraction of NACs is present in these datasets, we further seek to use microarray based on whole genome coverage to provide deep insights into the expression patterns of NACs. We have reanalyzed the *Populus *microarray data generated by Wilkins and coworkers [61]. Only six NAC genes (PNAC007, 018, 019, 052, 082 and 163) did not have their corresponding probe sets in the dataset, and the expression profiles of the rest of 157 NAC genes were analyzed as indicated in Figure 7. The majority of NAC genes showed distinct tissue-specific expression patterns across the nine tissues examined (Figure 7 and Additional file 9). It is notably that a relatively large fraction of NAC genes, accounting for more than 40% (67 of 163), were preferentially expressed in male and female catkins. Among these genes, thirty-four gene (50%) showed the highest transcript abundances in male catkins, twelve (18%) had the highest accumulation in female catkins, and the remaining 21 (32%) showed approximately equal transcript abundances in both male and female catkins. The number of NAC genes fell into this category is significantly higher compared to that of MYB transcription factor gene family analyzed by the same microarray data, in which, fifty (28%) R2R3-MYB genes showed the highest transcript accumulation in catkins [61]. The large proportion of NAC genes with the highest transcript abundance in catkins may probably attribute to the unique characteristic of sex determination in *Populus *lineage. The previous phylogenetic analysis revealed that these catkin-specific genes were dispersed among different subfamilies rather than divided into distinct subgroups (Figure 1).

Thirteen (8%) NAC genes showed the highest transcript abundances in differentiating xylems, including four xylem-specific NACs (PNAC070, 086, 121 and 122) identified in the previous microarray analysis (Figure 6C). The six well-characterized NAC genes involved in the secondary cell wall thickening in the previous studies were all included in this category [19]. The expression pattern of PNAC008 gene was inconsistent with the above microarray analysis (Figure 6). The current data revealed the highest expression of PNAC008 gene in young leaves and female catkins rather than in differentiating xylems. The number of NAC genes with the highest transcript abundances in differentiating xylems seems to be a little bit lower than that of R2R3 MYB gene family, which has 23 members accounting for about 12 percent of the total [61]. In addition, a considerable fraction of 24 (15%) NAC genes were specifically expressed in roots.

Interestingly, a subset of NAC genes demonstrated different expression patterns between dark-grown etiolated seedlings and continuous light grown seedlings, suggesting that these NACs may be subject to photoperiodic regulation. Strikingly, the majority of NAC genes did not show seemingly high abundances of transcript in either young or mature leaves as depicted by the previous ESTs digital profiles analysis (Figure 4 and Additional file 7). One possible explanation may lie in the fact of different sample size and coverage exemplified in the analyses.

The high proportion of segmental duplication of NAC genes and the preferential retention of duplicates raises the question about their functional redundancy. Duplicate genes may have different evolutionary fates: nonfunctionalization, neofunctionalization, or subfunctionalization [76], which may be indicated with divergence in expression patterns. Of the 49 homologous pairs of NAC genes, six genes (PNAC007, 018, 019, 052, 082 and 163) did not have corresponding probe sets on Affymetrix microarray and were excluded for further analysis. Therefore, the remaining 44 homologous pairs were analyzed. Among them, nineteen pairs were located onto duplicated blocks and three pairs were tandem duplicates. Five pairs (PNAC004/006, PNAC012/013, PNAC084/085, PNAC129/130 and PNAC144/145) out of 19 segmental duplications shared the same expression patters with respect to the tissues examined (Figure 7). In addition, the expression pattern of homologous pair PNAC004/006 was also supported by a broad sample sets from the previously microarray and ESTs profiles (Figure 4). Although the expression patterns of the rest of 14 duplicate genes were partially redundant, distinct pattern shifts can be discerned, which suggested that they have undergone subfunctionalization. These findings indicated that expression patterns of NACs have diverged quickly after segmental duplications, thus the NAC genes in *Populus *are likely to have been retained by substantial subfunctionalization. For example, PNAC008 gene were mainly expressed in young leaves and male catkins, whereas its duplicate counterpart PNAC010 gene extended to a broader expression patterns in young and mature leaves, female and male catkins (Figure 7).

Surprisingly, two homologous pairs (PNAC049/050 and PNAC121/122) also exhibited almost identical patterns of transcript accumulation but were located onto different duplication blocks (Figure 7). The reasons underlying their highly identical expression profiles remain to be investigated since we could not draw any clues from the segmental duplication events. The rest ten homologous pairs also showed almost identical expression patterns, but we could not locate them onto duplicated blocks currently since some of them were mapped to not-yet assembled scaffolds.

As for the tandem duplicated clusters with three genes, the expression patterns of two of them (PNAC020/022, PNAC033/035, PNAC141/143) were almost identical, while significant diversification was observed for the third member, which may indicate neofunctionalization. For example, tandem duplicate PNAC020 and PNAC022 were predominantly expressed in male catkins followed by roots, while PNAC030 was largely expressed in root followed by female catkin (Figure 7). As for the tandem duplicated clusters with two genes, their expression patterns diversified significantly, indicative of substantial neofunctionalization during subsequent evolution processes.

### Validation of NAC gene expressions by quantitative real-time RT-PCR

**Figure 8:**
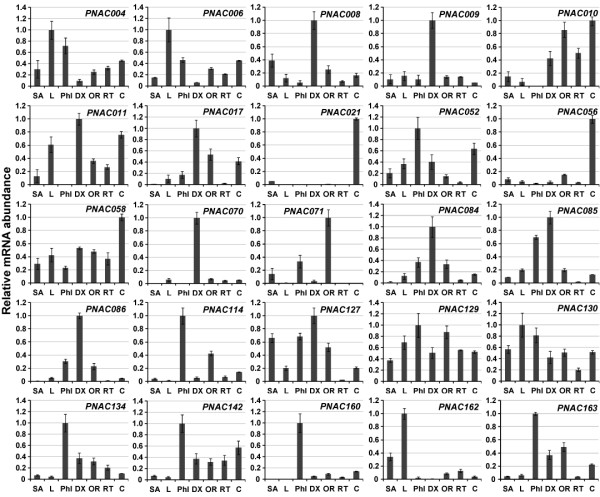
**Expression analysis of selected NAC genes using quantitative real-time RT-PCR (RT-qPCR)**. The relative mRNA abundance of 25 selected NAC genes was normalized with respect to reference gene UBQ10 in different tissues. The bars are standard deviations (SD) of three technical repeats. SA, shoot apices; L, leaf (4~6 internodes); Phl, phloem; DX, differentiating xylem; OR, old root; RT, root tip; C, cortex.

To verify the expression profiles of *Populus *NAC genes obtained by *in silico *EST and microarray data analysis, quantitative real-time RT-PCR (RT-qPCR) was performed on seven different tissues for 25 selected NAC genes. As illustrated in Figure 8, the genes showed very distinct tissue-specific expression patterns, which were in good agreement with the above microarray and EST profiles (Figure 6, 7). For example, in accordance with previous findings, a subset of eight genes among the 25 NACs tested were exclusively expressed in differentiating xylem, indicative of their putative roles in secondary cell wall formation. Three genes namely PNAC009 (PtWND4B), PNAC071 (PtWND1B) and PNAC085 (PtWND2B) have been functionally characterized in a recent report [19]. In the present study, PNAC071 gene was shown to primarily express in old root, while microarray profiles indicated the highest transcript abundance in differentiating xylems (Figure 7). Seven genes showed the highest transcript levels in phloem and four genes had the most abundant transcripts in cortex, which were both enriched in primary cell walls. These genes may have putative roles in primary cell wall biosynthesis and wood development. Consistent with the microarray profiles, four genes (PNAC004, 006, 130 and 162) were expressed specifically in leaves. Accordingly, PNAC004 gene had the largest number (up to 24) of corresponding EST sequences originated from leaves, which lent further supports to the above statement. All the 25 genes tested have constitutively weak expression levels in root meristem. Since only a few numbers of NAC genes have been functionally characterized up to date, our results presented here may provide some clues for the selection of candidate genes for further characterization.

## Conclusion

In the present study, a comprehensive analysis including phylogeny, chromosomal location, gene structure, conserved motifs, and expression profiling of NAC gene family in *Populus *was performed. We identified a sum of 163 full-length NAC genes in *Populus *genome, and they were phylogeneticly clustered into 18 distinct subfamilies. The exon/intron structure and motif compositions of NACs were highly conserved in each subfamily, indicative of their functional conservation. The NAC genes were non-randomly distributed across 19 LGs, and a high proportion of NACs were preferentially retained duplicates located on the duplicated blocks, which indicated that segmental duplications contribute significantly to the expansion of *Populus *NAC gene family. Although a majority of NAC genes showed specific temporal and spatial expression patterns based on EST frequency and microarray data analyses, the expression patterns of a considerable proportion of duplicate genes (14 of 44) were partially redundant, suggesting the occurrence of subfunctionalization during evolutionary process. In other words, a fraction of NAC genes in *Populus *are likewise to have been retained by substantial subfunctionalization. Furthermore, we identified a subset of *Populus *NAC genes with putative functional roles in wood-forming and secondary cell wall biosynthesis, and results presented here will be helpful for future functional studies to unravel their divergent roles.

## Methods

### Database search and sequence retrieval

The *P. trichocarpa *genome sequences were downloaded from Phytozome http://www.phytozome.net/poplar. Hidden Markov Model (HMM) profile of NAC domain (PF02365) downloaded from Protein family (Pfam) http://pfam.sanger.ac.uk/ was exploited for the identification of the NAC genes from *Populus *genome using HMMER (v 2.3.2) [89]. All non-redundant hits with expected values less than 1.0 were collected and then compared with the NAC family in PlnTFDB http://plntfdb.bio.uni-potsdam.de/v3.0/[3] and DPTF http://dptf.cbi.pku.edu.cn/[58]. As for the incorrectly predicted genes, manual reannotation was performed using online web server FGENESH http://linux1.softberry.com/berry.phtml[59]. The reannotated sequences were further manually analyzed to confirm the presence of NAM domain using InterProScan program http://www.ebi.ac.uk/Tools/InterProScan/[60]. Sequences of *Arabidopsis *and rice NAC domain proteins were downloaded from the *Arabidopsis *genome TAIR 9.0 release http://www.Arabidopsis.org/ and rice genome annotation database (http://rice.plantbiology.msu.edu/, release 5.0), respectively.

### Phylogenetic analysis

Multiple sequence alignments of the full-length protein sequences, including the highly conserved N-terminal NAM domain and the more divergent C-terminal activation domain, were performed by Clustal X (version 1.83) program [90]. The unrooted phylogenetic trees were constructed with MEGA 4.0 using the Neighbor-Joining (NJ), Minimal Evolution (ME) and Maximum Parsimony (MP) methods and the bootstrap test carried out with 1000 iterations [91]. Pairwise gap deletion mode was used to ensure that the more divergent C-terminal domains could contribute to the topology of the NJ tree.

### Genomic structure and chromosomal location

Gene structure display server (GSDS) program [92] was used to illustrate exon/intron organization for individual NAC genes by comparison of the cDNAs with their corresponding genomic DNA sequences from Phytozome http://www.phytozome.net/poplar. Identification of homeologous chromosome segments resulting from whole-genome duplication events was accomplished as described in Tuskan *et al*. (2006) [5]. Blocks of the same color represent the homologous chromosome segments. The tandem gene duplications in *Populus *were identified according to the same criteria described in rice [93]. Genes separated by five or fewer gene loci in a range of 100 kb distance were considered to be tandem duplicates.

### Identification of conserved motifs

The program MEME version 4.3.0 was used for the elucidation of motifs in 163 deduced *Populus *NAC protein sequences http://meme.sdsc.edu[94]. MEME was run locally with the following parameters: number of repetitions - any, maximum number of motifs - 20, and the optimum motif widths were constrained to between 6 and 200 residues. Structural motif annotation was performed using the SMART http://smart.embl-heidelberg.de[95] and Pfam http://pfam.sanger.ac.uk databases [96].

### EST profiling and microarray analysis

A total of 429,444 *Populus *EST sequences were downloaded from the GenBank database (http://www.ncbi.nlm.nih.gov/est/, release 121009). The coding regions of NAC genes were used as queries to perform a local BLASTN search against all of the ESTs. Matches above 96% identity and over an alignment of at least 100 bp were considered as corresponding sequences of the NAC genes. The entries identified were manually inspected for their tissue origin. In addition, the DigitalNorthen tool at the PopGenIE http://www.popgenie.org/ was utilized to produce the heat map with dendrograms of the NAC genes with their corresponding gene model IDs.

*Populus *microarray data were obtained from PopGenIE ftp://aspnas.fysbot.umu.se/, the expression data were gene-wise normalized and hierarchical clustered based on Pearson coefficients with average linkage in the Genesis (version 1.75) program [97].

The genome-wide microarray data performed by Wilkins and coworkers [61] were obtained from the NCBI Gene Expression Omnibus (GEO) [98] with accession number GSE13990. Probe sets corresponding to the putative *Populus *NACs were identified using an online Probe Match tool available at the NetAffx Analysis Center http://www.affymetrix.com/. For genes with more than one probe sets, the median of expression values were considered. When several genes have the same probe set, then they are considered to have same level of transcript abundance. the expression data were gene-wise normalized and hierarchical clustered based on Pearson coefficients with average linkage in the Genesis (version 1.75) program [97].

### Plant material and growth conditions

One-year-old *Populus deltoides *was grown in the greenhouse under long day conditions (16 h light/8 h dark) at a temperature 25°C~28°C. Shoot apices (internodes 1~3 from top, same as below), leaf (from internodes 4~6), developing xylem (from the basal internodes), phloem (from the basal internodes), cortex (from the basal internodes), old root and root tip (terminal 3~5 mm) tissues were separately harvested. All samples were immediately frozen in liquid nitrogen and stored at -80°C until required.

### RNA isolation and quantitative real-time RT-PCR (RT-qPCR)

Total RNA from the majority of the samples was extracted using TRIzol reagent (Invitrogen, Ca, USA) according to manufacturer's instructions. Alternatively, total RNA from cortex, old root and root tip were isolated by CTAB method [99] with minor modifications. RNA integrity was verified by 2% agar gel electrophoresis and SYBR Greenlstaining. Before cDNA synthesis, RNA was treated with RQ1 RNase-free DNase (Promega, Madison, WI, USA) according to the manufacturer's instructions to ensure no DNA contamination, and then the first-strand cDNA synthesis was carried out with approximately 2 μg RNA using the RevertAid First Strand cDNA Synthesis Kit (MBI, Fermentas) and oilgo-dT primers according to the manufacturer's procedure. Primers were designed using Beacon Designer v7.0 (Premier Biosoft International, Palo Alto, California, USA) with melting temperatures 58~60°C, primer lengths 20~24bp and amplicon lengths 51~199bp. Experimental details are given in additional file 10.

RT-qPCR was conducted on LightCycler^® ^480 Detection System (Roche, Penzberg, Germany) using SYBR Premix Ex Taq (TaKaRa, Toyoto, Japan). Reactions were prepared in a total volume of 20 μl containing: 2 μl of template, 10 μl of 2×SYBR Premix, 0.4 μl of each specific primer to a final concentration of 200 nM. The reactions were performed as the following conditions: initial denaturation step of 95°C for 10 s followed by two-step thermal cycling profile of denaturation at 95°C for 5 s, and combined primer annealing/extension at 60°C for 1 min for 40 cycles. No-template controls were included for each primer pair and each PCR reaction was completed in triplicate. To verify the specificity of the amplicon for each primer pair, a melting curve analysis was performed ranging from 60°C to 95°C with temperature increasing steps of 0.06°C/s (5 acquisitions per °C) at the end of each PCR run. Baseline and threshold cycles (Ct) were automatically determined using the LightCycler^® ^480 Software release 1.5.0. Relative gene expression with respect to internal reference gene UBQ10 was determined as described previously [100].

## Authors' contributions

RH, GQ and YK performed the computational analysis, expression analyses and drafted the manuscript cooperatively. DK and QG participated in the data mining, helped in *Populus *materials collection and data analysis. GZ conceived the project, supervised the analysis and critically revised the manuscript. All authors read and approved the final manuscript.

## Acknowledgements

This study was supported by grants from the National High-Tech Research and Development Program of China (to G.Z., 2009AA10Z101), the Program of 100 Distinguished Young Scientists of the Chinese Academy of Sciences (to G.Z., No. 428) and National Natural Science Foundation of China (No. 30901157).

## Supplementary Material

Additional file 1 **A complete list of NAC gene sequences identified in the present study**. The list comprises 163 NAC gene sequences identified. Amino acid sequences are deduced from their corresponding coding sequences and genomic DNA sequences are obtained from Phytozome http://www.phytozome.net/poplar, release 2.0. Click here for file

Additional file 2 **Phylogenetic tree of N-terminus NAC domain proteins from *Populus*, *Arabidopsis *and rice**. The unrooted tree was constructed using MEGA 4.0 with the Neighbor-Joining (NJ) method after alignment of the conserved N-terminus domain of 163 *Populus*, 105 *Arabidopsis *and 140 rice NAC genes. Only the tree topology is presented. Click here for file

Additional file 3 **Phylogenetic tree of full-length NAC domain proteins from *Populus*, *Arabidopsis *and rice**. The unrooted tree was constructed using MEGA 4.0 with the Neighbor-Joining (NJ) method after alignment of the full-length amino acid sequences of 163 *Populus*, 105 *Arabidopsis *and 140 rice NAC genes. Numbers at nodes indicate the percentage bootstrap scores and only bootstrap values higher than 50% from 1000 replicates are shown. The scale bar corresponds to 0.1 estimated amino acid substitutions per site. Click here for file

Additional file 4 **Microarray based expression profiles of *Arabidopsis *NAC genes across a variety of tissue/organs**. Expression of NAC genes during developmental stages are presented as heat maps generated using meta-analysis tool at GENEVESTIGATOR http://www.genevestigator.ethz.ch and clustered using hierarchical clustering with average linkage. The transcript levels are depicted by color scale representing log2 values. Dark blue denotes high expression and light blue denotes low expression. Click here for file

Additional file 5 **Pairwise identities between homologous pairs of NAC genes from *Populus***. Pairwise identities and sequence alignments of the 49 homologous pairs identified from *Populus *NACs. Click here for file

Additional file 6 **Exon/intron organization of *Populus *NAC genes**. Exons and introns are represented by green boxes and black lines respectively. The numbers indicate the splicing phases of the NAC genes, 0 refers to phase 0, 1 to phase 1, and 2 to phase 2. Click here for file

Additional file 7 **Sequence logos for the conserved motifs of *Populus *NAC domain proteins**. Conserved motifs and the sequence logos were generated using the MEME search tool. Numbers on the horizontal axis represent the sequence positions in the motifs and the vertical axis represents the information content measured in bits. Motif 2 represents the NAM sub-domain A, motif 5 represents the NAM sub-domain B, motif 1 represents the NAM sub-domain C, motif 3 represents the NAM sub-domain D, and motif 6 represents the NAM sub-domain E. Click here for file

Additional file 8 **Expression proflies of *Populus *NAC genes revealed by clustering analysis of the digital northern data**. The DigitalNorthen tool at the PopGenIE http://www.popgenie.org/ was utilized to produce the heat map with dendrograms of the NAC genes. Color bar at bottom represents the frequencies of EST counts. Click here for file

Additional file 9 **Raw data from microarray expression analyses for *Populus *NAC genes**. Sample abbreviations are defined in Figure 6 and Figure 7. Click here for file

Additional file 10 **Primer sequences of the selected NAC genes for RT-qPCR analysis**. A list of primer sequences of the 25 selected NAC gens for RT-qPCR assay. Click here for file
