# Supplementary figures and images for: Comprehensive Analysis of NAC Domain Transcription Factor Gene Family in Populus trichocarpa
Source: BMC Plant Biol. 2010 Jul 15;10:145. doi: 10.1186/1471-2229-10-145 (PMC3017804; doi:10.1186/1471-2229-10-145)

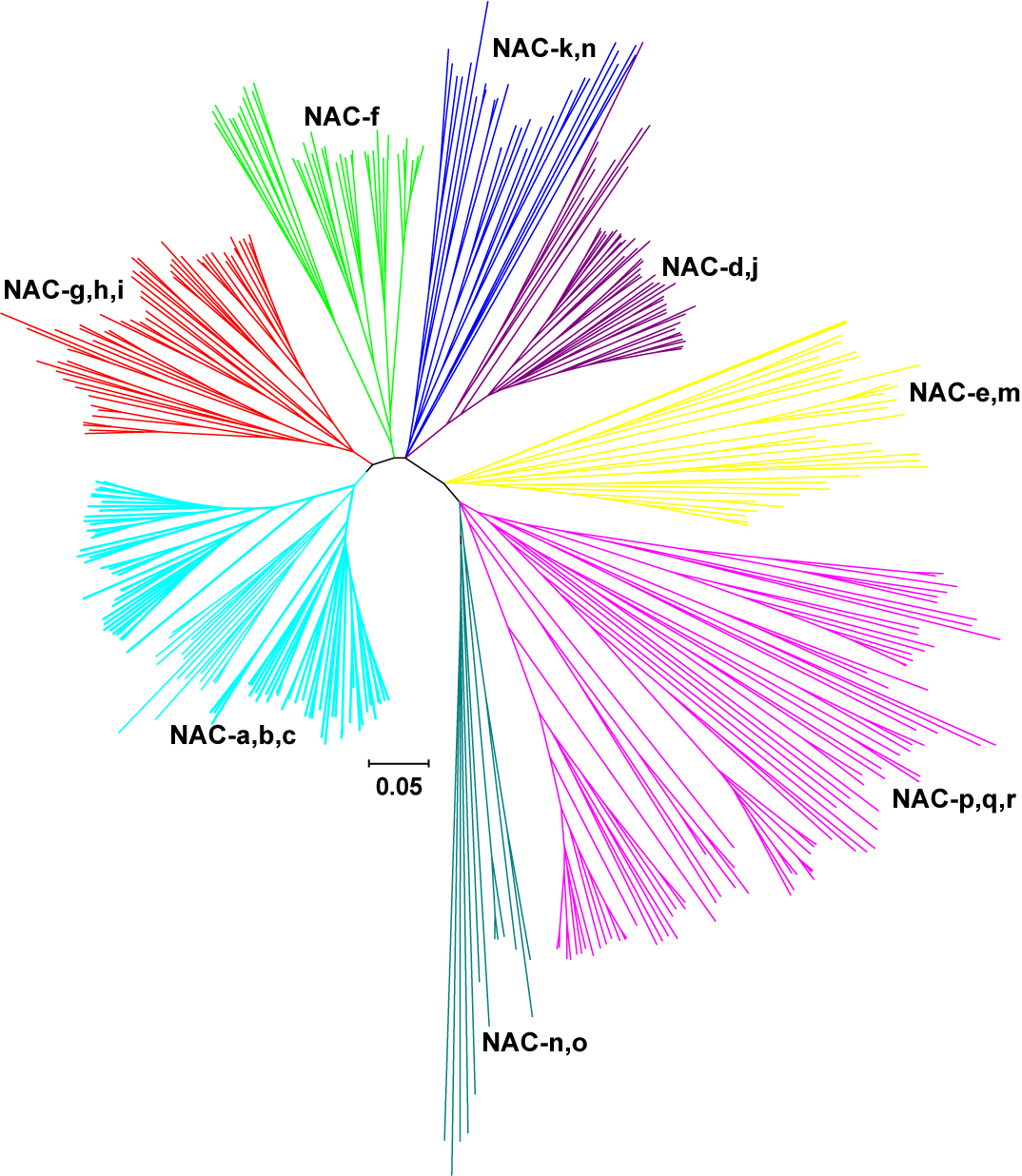

Supplement: Additional file 2 — Phylogenetic tree of N-terminus NAC domain proteins from Populus, Arabidopsis and rice. The unrooted tree was constructed using MEGA 4.0 with the Neighbor-Joining (NJ) method after alignment of the conserved N-terminus domain of 163 Populus, 105 Arabidopsis and 140 rice NAC genes. Only the tree topology is presented. [file 1471-2229-10-145-S2.JPEG]

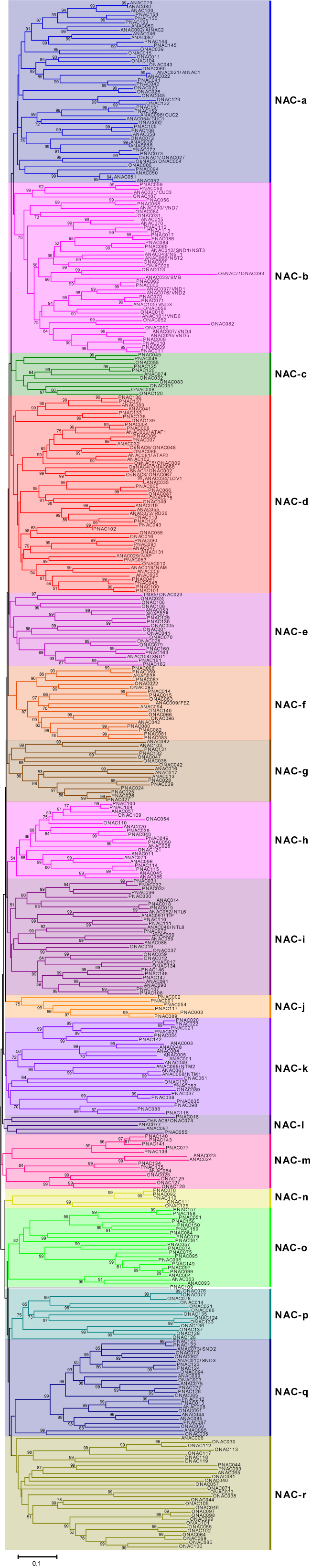

Supplement: Additional file 3 — Phylogenetic tree of full-length NAC domain proteins from Populus, Arabidopsis and rice. The unrooted tree was constructed using MEGA 4.0 with the Neighbor-Joining (NJ) method after alignment of the full-length amino acid sequences of 163 Populus, 105 Arabidopsis and 140 rice NAC genes. Numbers at nodes indicate the percentage bootstrap scores and only bootstrap values higher than 50% from 1000 replicates are shown. The scale bar corresponds to 0.1 estimated amino acid substitutions per site. [file 1471-2229-10-145-S3.JPEG]

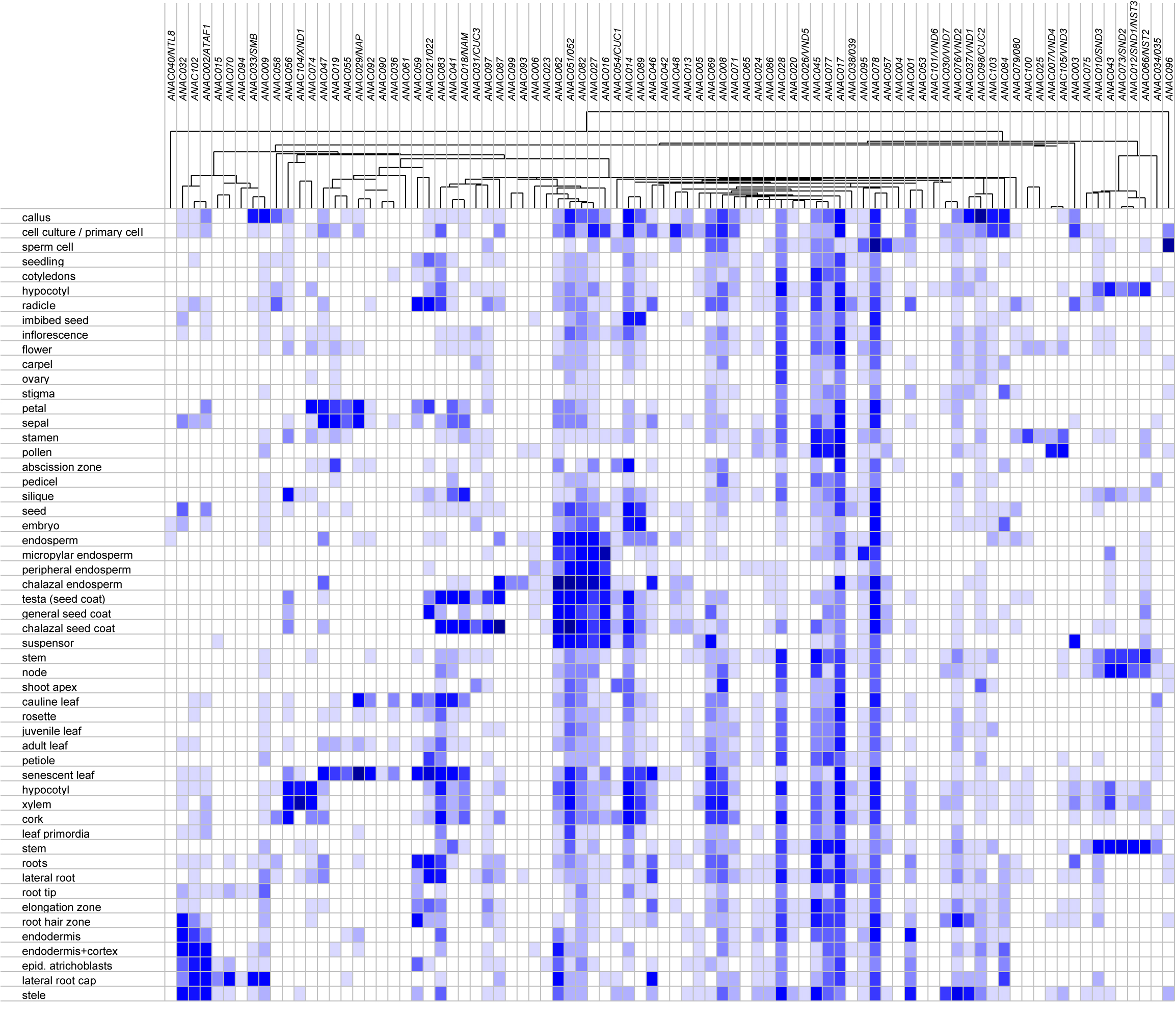

Supplement: Additional file 4 — Microarray based expression profiles of Arabidopsis NAC genes across a variety of tissue/organs. Expression of NAC genes during developmental stages are presented as heat maps generated using meta-analysis tool at GENEVESTIGATOR http://www.genevestigator.ethz.ch and clustered using hierarchical clustering with average linkage. The transcript levels are depicted by color scale representing log2 values. Dark blue denotes high expression and light blue denotes low expression. [file 1471-2229-10-145-S4.JPEG]

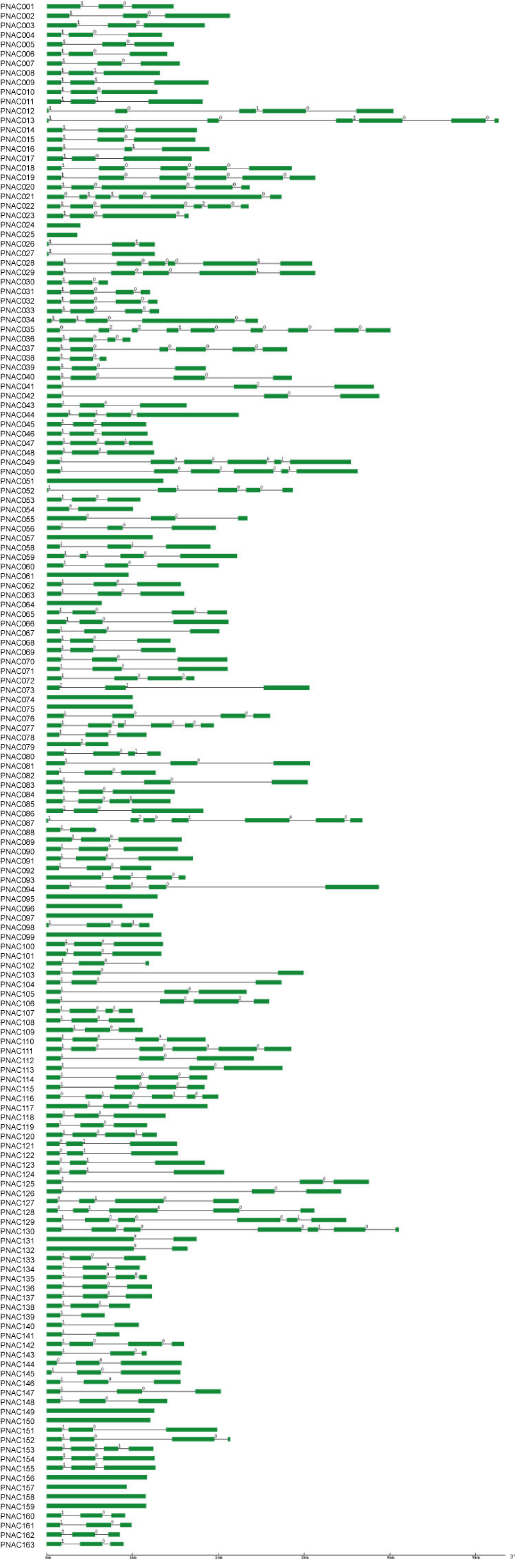

Supplement: Additional file 6 — Exon/intron organization of Populus NAC genes. Exons and introns are represented by green boxes and black lines respectively. The numbers indicate the splicing phases of the NAC genes, 0 refers to phase 0, 1 to phase 1, and 2 to phase 2. [file 1471-2229-10-145-S6.JPEG]

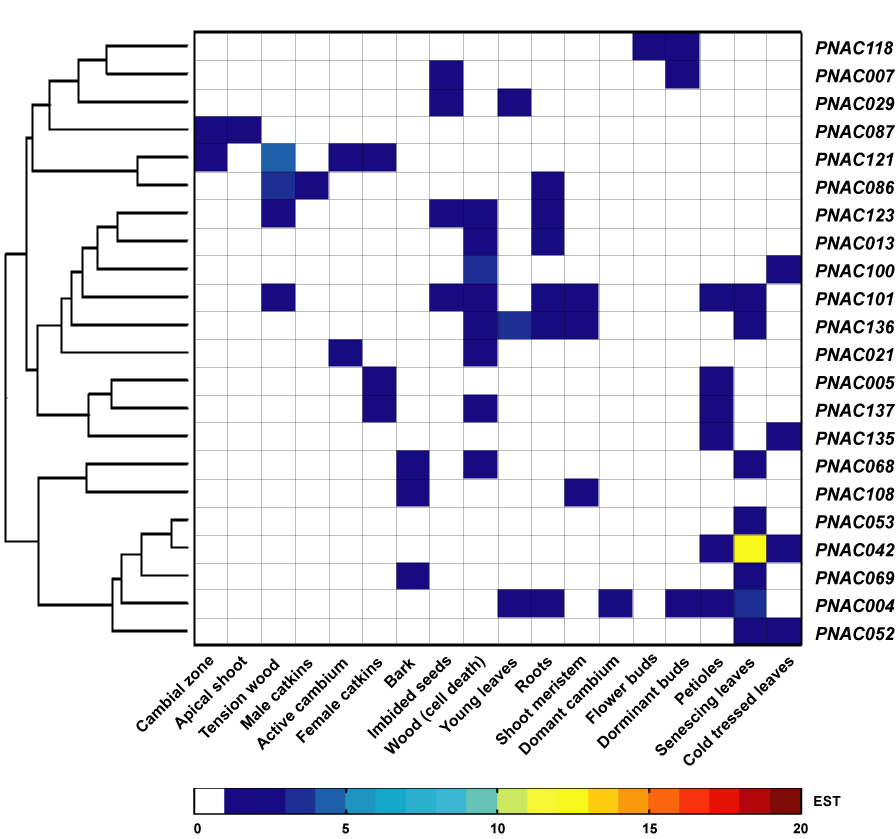

Supplement: Additional file 8 — Expression proflies of Populus NAC genes revealed by clustering analysis of the digital northern data. The DigitalNorthen tool at the PopGenIE http://www.popgenie.org/ was utilized to produce the heat map with dendrograms of the NAC genes. Color bar at bottom represents the frequencies of EST counts. [file 1471-2229-10-145-S8.JPEG]
